# Performance of the WID‐qEC test to detect uterine cancers in black women with abnormal uterine bleeding: A prospective observational cohort study in Ghana

**DOI:** 10.1002/ijc.35260

**Published:** 2024-12-10

**Authors:** Sebastian Ken‐Amoah, Elisa Redl, Bright K. S. Domson, James E. Barrett, Lena Schreiberhuber, Chiara Herzog, Rupali Arora, Allison Jones, Iona Evans, Dan Reisel, Esther Lamptey‐Mills, Vincent B. Nachinab, Theodora Pepera, Adeola Olaitan, Dorcas Obiri‐Yeboah, Patrick K. Akakpo, Martin Widschwendter

**Affiliations:** ^1^ Department of Obstetrics and Gynaecology University of Cape Coast Cape Coast Ghana; ^2^ European Translational Oncology Prevention and Screening (EUTOPS) Institute Hall in Tirol Austria; ^3^ Institute for Biomedical Aging Research University of Innsbruck Innsbruck Austria; ^4^ Department of Microbiology and Immunology University of Cape Coast Cape Coast Ghana; ^5^ Department of Pathology University College London London UK; ^6^ Department of Women's Cancer, UCL Elizabeth Garrett Anderson Institute for Women's Health University College London London UK; ^7^ Euracare Ghana Accra Ghana; ^8^ Department of Pathology University of Cape Coast Cape Coast Ghana; ^9^ General Hospital Hall, Tirol Kliniken Hall in Tirol Austria; ^10^ Department of Women's and Children's Health Karolinska Institutet Stockholm Sweden

**Keywords:** abnormal bleeding, black women, DNA methylation, endometrial cancer, ultrasound

## Abstract

The burden of uterine cancer is growing and, in the US and UK, mortality rates are poorest among black women. Early detection of these cancers is critical and poor performance of ultrasound in black women may contribute to adverse outcomes. Limited data on this topic are available from Africa. We assessed whether a simple DNA methylation test, the WID‐qEC, enables detection of all epithelial uterine (endometrial and cervical) cancers in women presenting with abnormal uterine bleeding (AUB) in Ghana. Among 118 women ≥40 years presenting with AUB, 106 consented to the study and a cervicovaginal sample was obtained for WID‐qEC testing. Subsequent to ultrasound assessment 102 women had a cervical or endometrial biopsy. Histopathology, ultrasound and WID‐qEC testing were analyzed and compared. Among the 102 volunteers, 8 and 15 were diagnosed with endometrial and cervical cancer (EC and CC), respectively. The receiver operating characteristic (ROC) area under the curve (AUC) was 0.56 (95% confidence interval [CI] 0.25–0.86) for sonographic endometrial thickness (ET) and 0.98 (95% CI 0.94–1.00) for the WID‐qEC test. Sensitivity and specificity of the prespecified ET ≥5 mm were 66.7% (95% CI 24.1–94.0) and 22.7 (95% CI 12.0–38.2) and for the prespecified WID‐qEC SUM‐PMR ≥ 0.3 were 100% (95% CI 56.1–100.0) and 76.1 (96%CI 60.9–86.9), respectively. In addition, 15 CCs were detected by the WID‐qEC test [sensitivity 100% (95% CI 74.7–100.0)]. The WID‐qEC test accurately detects both EC and CC in black women presenting with AUB.

## What's New?

Endometrial cancer (EC) is increasing significantly among women in low‐ and middle‐income countries (LMICs). EC detection may be improved by WID‐qEC testing, a DNA methylation‐based approach, though its use has been limited primarily to Caucasian populations. Here, the authors investigated the ability of WID‐qEC testing to detect endometrial and cervical cancer in black women in Ghana who presented with abnormal uterine bleeding. WID‐qEC testing successfully identified 100% of endometrial and cervical cancers in the study population. Utilizing simple sample collection and low‐cost, high‐throughput PCR‐based analysis, WID‐qEC is a promising tool for EC detection for all women in LMICs.

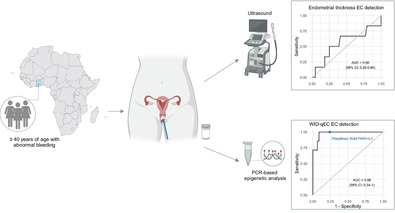

## 1 INTRODUCTION

Uterine cancers [endometrial and cervical cancers (ECs and CCs)] remain a significant global health concern, with varying rates of incidence and outcomes among different populations.^1^, ^2^, ^3^ Uterine cancers are the most common cancers, after breast cancer, with more than 1 million women diagnosed each year across the globe.^4^ In Ghana, women suffer a larger cancer burden than men (69.6% of all cases), with the commonest being breast (33.9%), cervix (29.4%), ovarian (11.3%) and endometrial (4.5%) cancers.^5^

CC is the most common gynecological cancer in low‐ and middle‐income countries (LMICs) and it remains the most common cause of death in women in 21 of the 48 countries in sub‐Saharan Africa.^6^ Its incidence is on the rise in these regions. In contrast to high‐income countries, where HPV vaccination and national CC screening programs are widely available, such resources are often lacking in LMICs, including those in sub‐Saharan Africa.^7^, ^8^, ^9^ In Ghana, most CC patients present with advanced stage disease.^10^ Consequently, a significant number of CC cases in these settings are diagnosed in women who present with abnormal uterine bleeding (AUB).

While EC is less common in LMICs, the burden of the disease in sub‐Saharan Africa is increasing. Indeed, the International Agency for Research on Cancer estimates that the incidence and mortality of EC will double in LMICs in the next two decades.^11^ EC, like CC, often presents with AUB. This overlapping presentation with AUB complicates timely and accurate diagnosis in these settings.

Current diagnostic pathways for EC are less reliable in black women and contribute to delayed interventions and poorer prognosis.^12^, ^13^ Data about the diagnostic challenges were largely generated in the US and UK, where the burden of EC in black women is also disproportionate: black women in the US and UK are more than twice as likely as white women to die from EC.^13^ Diagnostic challenges in black women presenting with AUB include the higher prevalence of uterine fibroids, which potentially lowers the sensitivity and specificity of sonographic assessment of the endometrium,^14^ and the higher risk of high‐grade ECs such as serous carcinoma and carcinosarcoma with poor prognoses,^15^ 25% of which are missed by ultrasound.^16^ Data from Ghana are limited but also show a higher prevalence of fibroids^17^; in addition, women with lower parity (<5 children) present with EC at an earlier stage than women with higher parity.^10^

Most women presenting with AUB in LMICs like Ghana will have an endometrial biopsy taken for histology and this incurs significant cost for the women. In addition, few experts in histological diagnosis are available. These factors, collectively, contribute to delayed diagnosis and hence there is an urgent need for a highly sensitive, cost‐effective, accurate and objective test to detect uterine cancers.

The WID‐qEC test, a novel diagnostic assay designed to detect uterine cancer of epithelial origin, shows promise based on recent studies which included mostly white women. This test, which assesses DNA methylation (DNAme) of two gene regions (*ZSCAN12* and *GYPC*) has demonstrated its ability to detect both endometrial^18^ and cervical^19^ cancers and outperforms sonography.^20^ However, its performance in black women with AUB remains unexplored. To fill this critical knowledge gap and comprehensively characterize the performance and applicability of the test in various populations, data from diverse groups of women are needed.

This study assessed the accuracy (sensitivity and specificity) of the WID‐qEC test in detecting uterine cancers in women, aged ≥40 years, presenting with AUB at the Cape Coast Teaching Hospital and its affiliated centers in Ghana.

## 2 METHODS

### 2.1 Study conduct and study population

This was a prospective, consecutive observational cohort study conducted at the Cape Coast Teaching Hospital (CCTH) in Ghana and its affiliated health facilities from June 1, 2023 to December 31, 2023. All women aged ≥40 years who attended the facilities due to AUB were invited to participate. The affiliated health facilities that took part in the study are the University of Cape Coast Hospital, Cape Coast, Our Lady of Grace Hospital, Breman Assikuma, Swedru Government Hospital, Swedru, St Francis Xavier Hospital, Assin Fosu, Ahmadiyya Muslim Hospital, Daboase and Effia Nkwanta Regional Hospital, Sekondi. At enrollment and prior to any procedures, a cervicovaginal smear was obtained, stored and analyzed in one batch after recruitment was completed. All women provided baseline data (age and ethnicity; menopausal status, height and weight were only recorded in <50% of volunteers).

The results reported herein follow the Strengthening the Reporting of Observational Studies in Epidemiology (STROBE) guidelines for cohort studies.^21^

### 2.2 Sample collection

A standard Cusco speculum was used to access and visualize the cervix prior to any other interventions. A cervical sample was obtained using a Cervex‐brush (Rovers Medical Devices) and immersed and rinsed in a SecurTrainer™ specimen container (Simport) containing 10 mL of PreservCyt solution labeled with a unique Sample ID barcode.

### 2.3 Standard diagnostic pathway

All women were examined and underwent a standard ultrasonographic assessment. In addition to endometrial thickness (ET), the presence of fibroids was recorded. All women were recommended a histological assessment to enable a comprehensive clinical evaluation. In the outpatient clinic, all women with lesions on the cervix had a punch biopsy. Endometrial samples were obtained by dilation and curettage (D&C) in women whose source of bleeding was not a cervical lesion.

### 2.4 Histopathological assessment

All tissue samples were fixed in 10% buffered formalin and transported to the pathology laboratory, where all the tissue specimens were processed and sectioned for examination by the pathologist. All malignant cases were reported according to the World Health Organization 2023 guidelines (https://tumourclassification.iarc.who.int/chapters/34) and entered into an electronic medical record system that allowed data extraction for analysis. Endometrial hyperplasia was categorized according to the recent two‐tier system. All discordant cases were reviewed by an NHS‐UK consultant pathologist.

### 2.5 DNA methylation analysis

DNA was isolated from collected sediment as described previously.^20^ The WID‐qEC test, which utilizes quantitative real‐time PCR on bisulfite‐modified DNA to assess DNAme (MethyLight), was performed as previously described.^20^ *GYPC* and *ZSCAN12* percentages of fully methylated reference (PMR) values were calculated. WID‐qEC results were reported as the SUM‐PMR of both target PMRs.

### 2.6 Data handling

All demographic and clinical data, including performed medical procedures (i.e., ultrasound) and their results, were collected before any test outcome data became available. Histological outcome data were recorded over a 1‐month period after recruitment ended and the database was locked on February 1, 2024. The 106 cervicovaginal samples were stored at room temperature before being shipped to the University of Innsbruck for WID‐qEC test analysis. Laboratory staff were blinded to clinical parameters and clinical outcomes.

### 2.7 Study outcomes

The primary outcome was to assess the performance of the WID‐qEC test utilizing the prespecified cutoff (≥0.3 WID‐qEC SUM‐PMR^20^) and computing the area under the receiver operating characteristic (ROC) curve (AUC) using the numerical values of both parameters to predict the final histological diagnosis (i.e., invasive uterine cancer vs. no cancer).

### 2.8 Statistical methods

A sample size of 200 was determined to be able to detect a significant difference in the ultrasound‐estimated positive predictive value of 5.8% and WID‐qEC‐estimated positive predictive value of 22.2% at a power of 75% and an alpha level of 5%, using a single sample binomial comparison. All statistical analyses were carried out in R version 4.3.2. Comparisons between groups were made using the *t*.test function in the stats R package, version 4.3.2. ROC curves, areas under the curve, and corresponding 95% confidence intervals (CIs) were generated using the pROC package, version 1.18.2. Sensitivity and specificity, including 95% CIs were calculated according to the Wilson method using the prop.test function in the Rstats package, version 4.3.2.

### 2.9 Protocol deviations

Due to local needs and the higher risk of women being lost to follow‐up or needing multiple visits, tissue samples were obtained, and histological assessment was done for all patients. Cytology was not routinely done because women presented with AUB and required adherence to standard treatment guidelines for managing such scenarios. The gold standard, histopathological assessment of tissue, was thus adopted for all participants. The original intention was to recruit 200 women, but we ended the study after recruitment of 106 patients as a higher‐than‐expected number of cancer cases were observed within the cohort.

## 3 RESULTS

**FIGURE 1:**
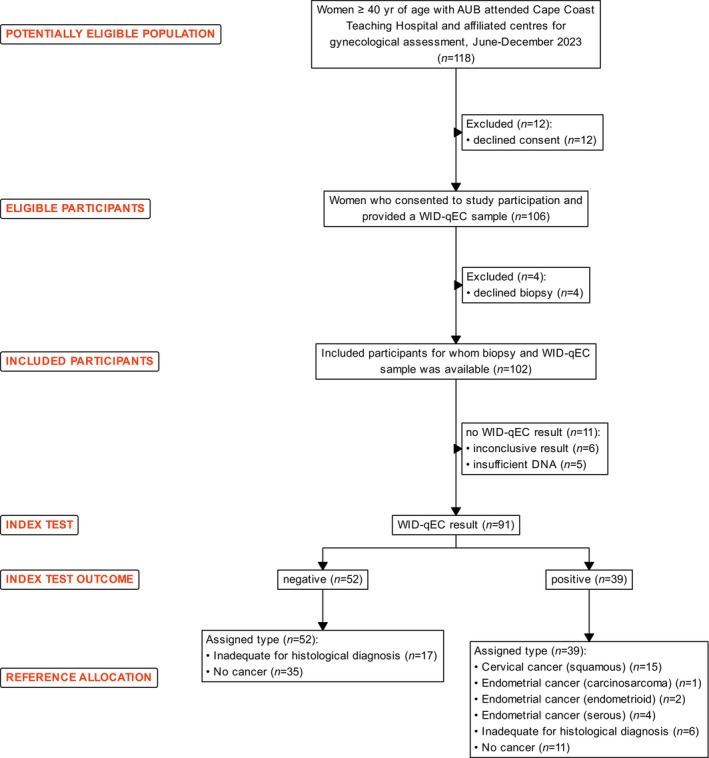
Study population. AUB, abnormal uterine bleeding.

Between June and December 2023, a total of 118 eligible patients ≥40 years attended the Cape Coast Teaching Hospital and affiliated health facilities in Ghana due to AUB. The total number of eligible patients may potentially have been higher, but not all patients were registered. 106/118 (89.8%) consented to participate in the study and in 102/106 (96%) women a biopsy from the cervix or the endometrium was obtained for histological assessment (Figure 1). All patients were of black African ethnicity.

Among the 102 patients for whom a histological assessment was obtained, 23 (22.5%) were diagnosed with cancer: eight (7.8%) with EC and 15 (14.7%) with CC. Fifty‐three (52.0%) were deemed to be cancer‐free. In 26 (25.5%) patients, the histological sample obtained was inadequate to make a definitive diagnosis (Figure 1). Four of these patients with inconclusive test results opted not to have a repeat biopsy; 15 opted for hysterectomy but refused histopathology evaluation of hysterectomy specimen due to cost by not delivering the specimen to the histopathology laboratory (in Ghana, it is the responsibility of patients to deliver their own specimen to the histopathology laboratory); 7 were lost to follow‐up.

The characteristics of the studied cohort are outlined in Table 1. The average age was 53, with 48 (47.1%) patients aged 50 years or older. ET was measurable in 82 patients; in 13 patients, the endometrium could not be visualized and seven patients refused the assessment of their endometrium. Fifty‐three (52.0%) patients were diagnosed with fibroids. Among all 102 patients, 86 (84.3%), 15 (14.7%) and 1 (1.0%) had their histology based on a specimen obtained by curettage, punch biopsy or hysterectomy, respectively.

**TABLE 1:** Characteristics of the study population.

Characteristic	All (*N* = 102)	EC (*N* = 8)	CC (*N* = 15)	No cancer (*N* = 53)	Inconclusive (*N* = 26)
	No.	No.	No.	No.	No.
Age (years)					
40–49	54	0	5	37	12
≥50	48	8	10	16	14
ET (all women) (mm)					
<3	6	1	1	2	2
≥3 but <5	13	1	0	8	4
≥5	63	4	10	34	15
Not measurable	20	2	4	9	5
ET (women ≥50 years only) (mm)					
<3	3	1	0	2	0
≥3 but <5	4	1	0	1	2
≥5	30	4	7	11	8
Not measurable	11	2	3	2	4
Fibroids (sonographically)					
Present	53	3	4	30	16
Absent	33	4	8	17	4
Not determined	16	1	3	6	6
Mode of biopsy					
Curettage	86	7	4	52	23
Punch	15	1	10	1	3
Hysterectomy	1	0	1	0	0
Histology					
Squamous cell carcinoma of the cervix	15	0	15	0	0
Endometrioid endometrial cancer	3	3	0	0	0
Serous endometrial cancer	4	4	0	0	0
Carcinosarcoma of endometrium	1	1	0	0	0
Benign endometrial hyperplasia/polyp	30	0	0	29	1
Atypical hyperplasia	1	0	0	1	0
Inflammatory changes	14	0	0	13	1
Cervical Intraepithelial Neoplasia (CIN)	3	0	0	1	2
Pregnancy products	4	0	0	3	1
Other benign cervical changes	5	0	0	3	2
Leiomyoma	2	0	0	2	0
Leiomyosarcoma	1	0	0	0	1
Sonographically ovarian cancer suspected	1	0	0	1	0
Non‐diagnostic	18	0	0	0	18
Grade					
1	2	1	1	0	0
2	10	0	10	0	0
3	11	7	4	0	0
WID‐qEC SUM‐PMR					
<0.3	52	0	0	35	17
≥0.3	39	7	15	11	6
Inadequate	11	1	0	7	3

Abbreviations: CC, cervical cancer; EC, endometrial cancer; ET, endometrial thickness; PMR, percentage of fully methylated reference.

All 15 (100%) CCs were squamous cell carcinoma‐NOS, and three (37.5%), four (50.0%) and one (12.5%) of the ECs were endometrioid, serous and carcinosarcoma, respectively. Almost all of the ECs (7/8; 87.5%) and 4/15 (26.7%) of the CCs were grade three/high‐grade cancers. Information on pathological stage was not available, because the specimens that formed the basis of histopathological diagnosis were curettages or punch biopsies. Clinical stage was also not available at the conclusion of this study because clinical staging is carried out as part of the work‐up to decide the definitive management modality.

**FIGURE 2:**
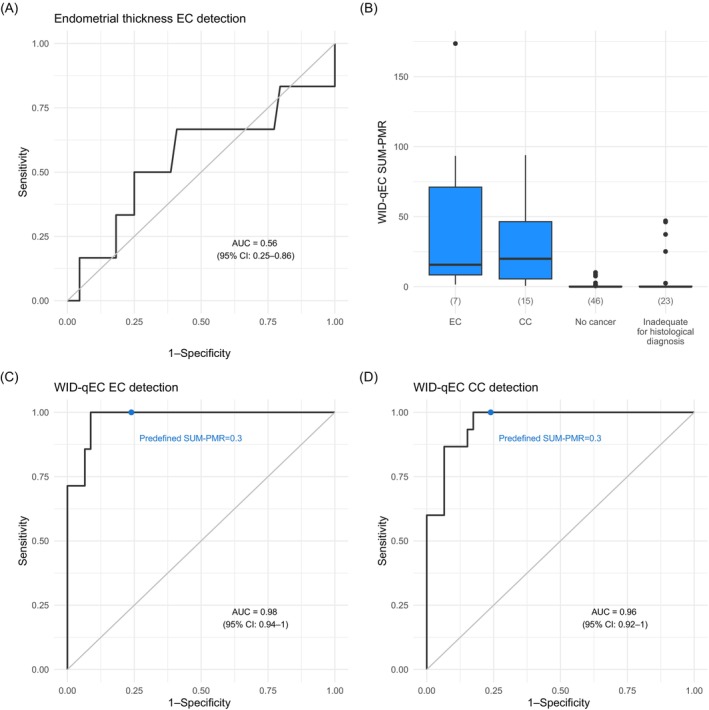
Performance of sonography and WID‐qEC test to detect uterine cancers. (A) ROC for endometrial thickness assessments using sonography was calculated based on the entire study population excluding cases with cervical cancer. (B) Boxplots depict WID‐qEC SUM‐PMR results for all assessed samples. ROCs for WID‐qEC detecting (C) EC or (D) CC were calculated based on the entire study population excluding cases with cervical cancer in (C) and endometrial cancer cases in (D). AUC, area under the curve; CC, cervical cancer; EC, endometrial cancer; PMR, percentage of fully methylated reference.

Among all 82 women with measurable ET, 63 (76.8%) had an ET ≥ 5 mm. Out of the 37 women ≥50 years with a measurable ET, 30 (81.1%) had an ET ≥ 5 mm. Among the eight ECs, four (50.0%) cancers were not detectable by ultrasound when applying a 5 mm cutoff (in two serous cancers the endometrium could not be visualized and in one carcinosarcoma and one endometrioid cancer the ET was <5 mm). The ROC AUC for ET was 0.56 (95% CI: 0.25–0.86) (Figure 2A) and only slightly changed when applying to women ≥50 years [0.60 (95% CI: 0.26–0.94)] (Figure S1A). Applying 3 and 5 mm ET cutoffs led to sensitivities and specificities (Table 2) that were unacceptably low for all women.

**TABLE 2:** Performance characteristics of the ultrasound and WID‐qEC test for EC detection on women with a final diagnosis.

	ET ≥ 3 mm		ET ≥ 5 mm		SUM‐PMR ≥ 0.3	
	No./total no.	% (95% CI)	No./total no.	% (95% CI)	No./total no.	% (95% CI)
Sensitivity	5/6	83.3 (36.5–99.1)	4/6	66.7 (24.1–94.0)	7/7	100.0 (56.1–100.0)
Specificity	2/44	4.5 (0.8–16.7)	10/44	22.7 (12.0–38.2)	35/46	76.1 (60.9–86.9)
PPV	5/47	10.6 (4.0–23.9)	4/38	10.5 (3.4–25.7)	7/18	38.9 (18.3–63.9)
NPV	2/3	66.7 (12.5–98.2)	10/12	83.3 (50.9–97.1)	35/35	100 (87.7–100)

Abbreviations: EC, endometrial cancer; ET, endometrial thickness; NPV, negative predictive value; PMR, percentage of fully methylated reference; PPV, positive predictive value.

The WID‐qEC test could be performed in 91/102 (89%) patients. Of the 11 samples that did not lead to a conclusive WID‐qEC result, 6/11 (54.6%) samples did not yield sufficient DNA and 5/11 (45.4%) samples showed inconclusive target Cq values in technical qPCR replicates with one replicate above the limit of detection. In a clinical setting, resampling would be required for these 11 women.

Comparing WID‐qEC levels in all 22 cancer cases that had WID‐qEC test results available with either all cancer‐free women or all women without a cancer diagnosis (i.e., cancer‐free and women without adequate histology combined) showed highly significant differences (*p* <.001) (Figure 2B). The respective ROC AUCs were 0.97 (95% CI: 0.93–1.00) when comparing all cancers to cancer‐free and 0.94 (95% CI: 0.89–0.98) when comparing to all women without a cancer diagnosis (Figure S2).

The ROC AUC for the WID‐qEC was 0.98 (95% CI: 0.94–1.00) (Figure 2C) and only slightly changed when applied to women ≥50 years [0.95 (95% CI: 0.87–1.00)] (Figure S1B). Based on the prespecified SUM‐PMR ≥0.3, the sensitivity of the WID‐qEC to detect EC was 100% at a specificity of 76.1% (Table 2). Among the 11 women with false positive results, the majority (6/11; 54.5%) were ≥50 years old and eight (72.2%), two (18.2%) and one (9.1%) participant had histological findings of hyperplasia, inflammatory changes and molar pregnancy products, respectively. The median ET in these women was 12.1 mm.

Based on the prespecified SUM‐PMR ≥ 0.3, the sensitivity of the WID‐qEC to detect CC was 100% at a specificity of 76.1% (Table 3). The respective ROC AUC (comparing CC to cancer‐free women) was 0.96 (95% CI: 0.92–1.00) (Figure 2D).

**TABLE 3:** Performance characteristics of the WID‐qEC test for CC detection on women with a final diagnosis.

	SUM‐PMR ≥ 0.3	
	No./total no.	% (95% CI)
Sensitivity	15/15	100.0 (74.7–100.0)
Specificity	35/46	76.1 (60.9–86.9)
PPV	15/26	57.7 (37.2–76.0)
NPV	35/35	100 (87.7–100.0)

Abbreviations: CC, cervical cancer; NPV, negative predictive value; PMR, percentage of fully methylated reference; PPV, positive predictive value.

## 4 DISCUSSION

Here, we have demonstrated that the WID‐qEC test identifies 100% of women with uterine cancers (i.e., endometrial and cervical cancers) among all women presenting with AUB in clinics in the Western and Central regions of Ghana. Our data also confirm the poor performance (predictability of endometrial carcinoma) of the subjective sonographic ET measurement compared to the objective WID‐qEC test in this population, which shows the same prevalence of EC when compared to other countries in Africa^22^ and other continents.^23^

Although the WID‐qEC sensitivity for EC detection is 100%, the specificity is somewhat lower than in our previous studies.^20^ Eight of the 11 women with false positive WID‐qEC results had hyperplasia on histologic assessment. Distinguishing hyperplasia and early stage or type one ECs is challenging and requires an adequate tissue sample or excision of the entire uterus for thorough examination. The presence of uterine fibroids may prevent adequate sampling of the relevant lesion by obstructing the uterine cavity. Despite curettage being the gold standard for sampling to rule out cancer in women presenting with AUB, a recent review and meta‐analysis of studies related to the use of endometrial biopsy for the diagnosis of endometrial carcinoma reveal its rather limited sensitivity of 78%.^24^ These data are also in line with our most recent study in which 2/11 cancers (18%) were missed by endometrial biopsy.^20^ Hence, it is possible that some of the 11 women with a positive WID‐qEC result who were assessed as cancer‐free based on curettage may have been diagnosed with a cancer had a hysterectomy been performed. This hypothesis is supported by the fact that the majority of these women had a high ET. It can be assumed that the thicker the endometrium, the more difficult it becomes to obtain sufficiently representative tissue, especially in the presence of fibroids.

Data from this cohort of black women confirm that the WID‐qEC is also able to detect 100% of invasive CCs. These findings are in line with our previous data from a case/control set using samples collected in Austria.^19^ Therefore, the WID‐qEC may also have a role in triaging patients with lesions at colposcopy in lower resource settings. Currently, all colposcopy patients require biopsy and histopathological analysis and patients at risk of CC are lost to follow‐up due to the costs associated with the procedure. The high sensitivity of the WID‐qEC may, therefore, be used to select patients who otherwise mandatorily must have at least a punch biopsy or a Loop Electrosurgical Excision Procedure (LEEP) to rule out cancer of the cervix when a concerning lesion is seen.

This is the first study which assessed the performance of a molecular test to detect endometrial and cervical cancers among black women presenting with AUB.

Our study has some limitations. Firstly, it lacks information on menopausal status, HRT use (which is low in this demographic), and cancer stage. However, this limitation is unlikely to impact our findings that the WID‐qEC test accurately detects uterine cancers in women presenting with AUB in clinics in Ghana. Secondly, the WID‐qEC test was inconclusive in one of the endometrial cancer cases due to sampling‐related issues; in a clinical setting, resampling would have been required. Finally, cytology was not obtained in this cohort meaning that WID‐qEC test results could not be correlated with cervical cytology results.

The WID‐qEC test could change the pathway of women presenting with abnormal bleeding. The study demonstrates that an ultrasound scan is an unreliable triage tool in black women. The same findings are independently confirmed in a recent paper by Doll et al., published while this manuscript was under review.^25^

The WID‐qEC test is highly sensitive, objective and is not operator‐dependent. In principle it also allows patients to self‐sample (previous data have demonstrated the WID‐qEC test can also be assessed on self‐collected samples^18^). Due to the very high sensitivity and specificity of the test, women with a negative WID‐qEC test result do not need a biopsy. The WID‐qEC test will be cheaper than the combination of ultrasound, the procedure of taking a biopsy (mainly D&C), and histological assessment. A large proportion of women can therefore be reassured without having to undergo and pay for the current gold standard.

Women with a positive WID‐qEC test result should ideally undergo a D&C under general anesthesia in order to obtain a good sample for histological assessment. This is particularly important because endometrial biopsies miss between 12% and 22% of cancers, when compared with histology obtained from the hysterectomy specimen.^24^ The fact that the WID‐qEC also detected 100% of cervical cancer makes this test particularly useful in countries without established cervical cancer screening where abnormal bleeding from the uterus or vagina are difficult to distinguish and are equally likely to be related to an endometrial or cervical malignancy.

Overall, the WID‐qEC is able to identify black women with endometrial or cervical cancer who present with abnormal bleeding. The test is based on a sample that can be simply obtained by a non‐expert and analyzed by an objective, low‐cost, high‐throughput PCR‐based test system that can return results quickly. Barriers to implementation of the WID‐qEC in clinical practice in a wide variety of environments—including resource constrained settings—are now low.

## AUTHOR CONTRIBUTIONS

**Sebastian Ken‐Amoah:** Conceptualization; methodology; writing – review and editing. **Elisa Redl:** Investigation; writing – review and editing. **Bright K. S. Domson:** Investigation; data curation. **James E. Barrett:** Formal analysis; writing – review and editing. **Lena Schreiberhuber:** Formal analysis; investigation; writing – review and editing. **Chiara Herzog:** Formal analysis; writing – review and editing. **Rupali Arora:** Methodology; writing – review and editing. **Allison Jones:** Methodology; writing – review and editing. **Iona Evans:** Methodology; writing – review and editing. **Dan Reisel:** Methodology; writing – review and editing. **Esther Lamptey‐Mills:** Methodology; writing – review and editing. **Vincent B. Nachinab:** Investigation; data curation; methodology; writing – review and editing. **Theodora Pepera:** Conceptualization; writing – review and editing. **Adeola Olaitan:** Conceptualization; writing – review and editing. **Dorcas Obiri‐Yeboah:** Methodology; writing – review and editing. **Patrick K. Akakpo:** Investigation; methodology; writing – review and editing; data curation. **Martin Widschwendter:** Conceptualization; formal analysis; writing – original draft; writing – review and editing; funding acquisition.

## FUNDING INFORMATION

Funded by the Samira Empowerment and Humanitarian Projects (Ghana), University College London Global Engagement Fund, The Land Tirol (EUTOPS Institute) and The Eve Appeal.

## CONFLICT OF INTEREST STATEMENT

J.E.B., C.H., E.R., A.J., I.E. and M.W. are inventors on WID‐qEC related patent applications. J.E.B., C.H. and M.W. are shareholders of Sola Diagnostics GmbH which holds own and licensed IP protecting commercialization of the WID‐qEC test. E.R. is an employee of Sola Diagnostics GmbH. The other authors have no conflict of interest to declare.

## ETHICS STATEMENT

The study was approved by the Cape Coast Teaching Hospital Ethical Review Committee (ref: CCTHERC/EC/2022/151) and the Innsbruck Medical University Ethical Committee (ref: 1411/2020). Written informed consent was obtained from all subjects. The study was registered (Trial Registration Number: ISRCTN18027281; https://doi.org/10.1186/ISRCTN18027281).

## Supporting information


Data S1.

Table S1.

## Data Availability

All the data that support the findings of this study are available in Table S1. Further information is available from the corresponding author upon request.
