# Supplementary material for: Performance of the WID‐qEC test to detect uterine cancers in black women with abnormal uterine bleeding: A prospective observational cohort study in Ghana
Source: Int J Cancer. 2024 Dec 10;156(5):1055–64. doi: 10.1002/ijc.35260 (PMC11701417; doi:10.1002/ijc.35260)
Supplement: Supplementary file 1 — Data S1. [file IJC-156-1055-s001.pdf]

Supplementary Appendix

Performance of the WID-qEC test to detect uterine cancers in black women with abnormal uterine bleeding: a prospective observational cohort study in Ghana

AUTHORS

Sebastian Ken-Amoah, Elisa Redl, Bright K S Domson, James E Barrett, Lena Schreiberhuber, Chiara Herzog, Rupali Arora, Allison Jones, Iona Evans, Dan Reisel, Esther Lamptey-Mills, Vincent B Nachinab, Theodora Pepera, Adeola Olaitan, Dorcas Obiri-Yeboah, Patrick K Akakpo and Martin Widschwendter

Supplementary table

Table S1. Phenotypic data of the study population. 3

Supplementary figures

Figure S1. Performance of sonography and WID-qEC test to detect EC in women ≥ 50 years of age. 4

Figure S2. Performance of WID-qEC test to detect uterine cancer cases compared to (A) cancer-free women or (B) all women without a cancer. 5

Table S1. Phenotypic data of the study population. File is separately available.

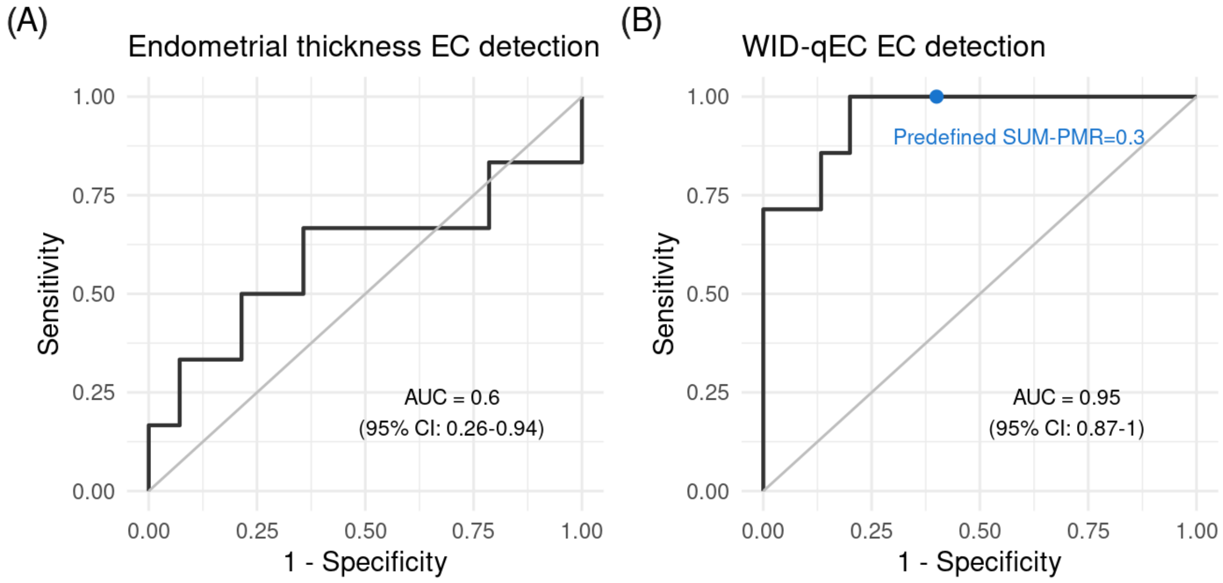

Figure S1. Performance of sonography and WID-qEC test to detect EC in women ≥ 50 years of age. ROCs for (A) endometrial thickness assessments using sonography and for (B) WID-qEC testing were calculated based on all women ≥ 50 years of age from the study population. AUC denotes area under the curve, EC endometrial cancer, PMR percentage of fully methylated reference.

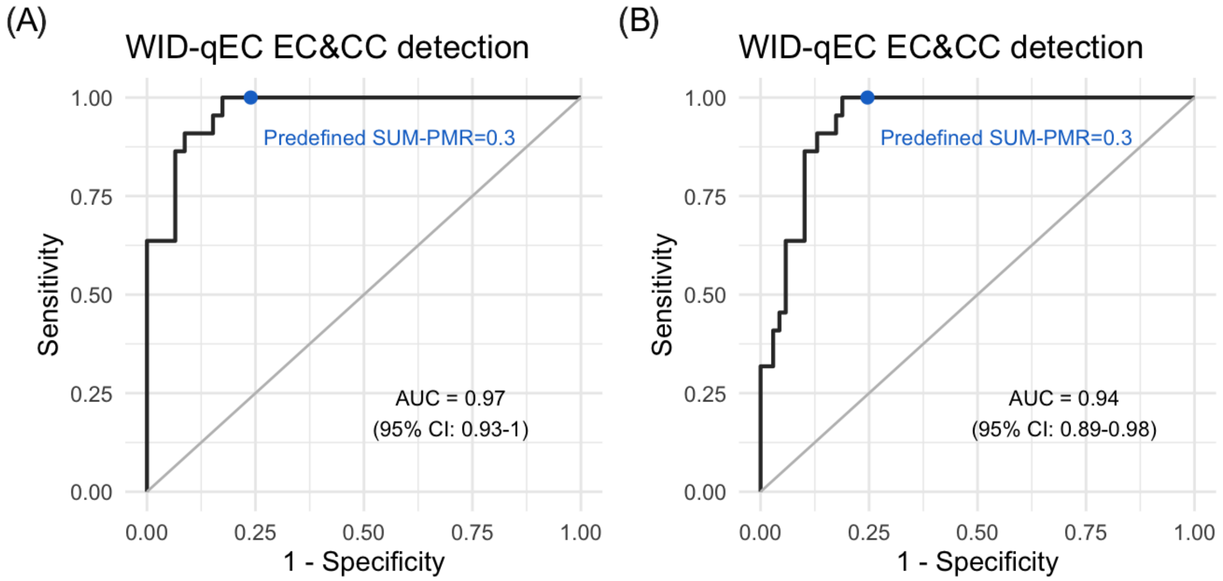

Figure S2. Performance of WID-qEC test to detect uterine cancer cases compared to (A) cancer-free women or (B) all women without a cancer. All women without a cancer were defined as the subpopulation which consisted of cancer-free women and women without adequate histology. Performance was assessed with ROCs. AUC denotes area under the curve, CC cervical cancer, EC endometrial cancer, PMR percentage of fully methylated reference.
